# A methodological framework for characterizing fish swimming and escapement behaviors in trawls

**DOI:** 10.1371/journal.pone.0243311

**Published:** 2020-12-11

**Authors:** Marianne Robert, Aurore Cortay, Marie Morfin, Julien Simon, Fabien Morandeau, Jean Louis Deneubourg, Benoit Vincent

**Affiliations:** 1 Ifremer, LTBH (Laboratory of Fisheries Technology and Biology), Lorient, France; 2 Center for Nonlinear Phenomena and Complex Systems, Université libre de Bruxelles, Bruxelles, Belgium; Universidad de Cádiz, Facultad de Ciencias del Mar y Ambientales, SPAIN

## Abstract

Knowledge about fish behavior is crucial to be able to influence the capture process and catch species composition. The rapid expansion of the use of underwater cameras has facilitated unprecedented opportunities for studying the behavior of species interacting with fishing gears in their natural environment. This technological advance would greatly benefit from the parallel development of dedicated methodologies accounting for right-censored observations and variable observation periods between individuals related to instrumental, environmental and behavioral events. In this paper we proposed a methodological framework, based on a parametric Weibull mixture model, to describe the process of escapement attempts through time, test effects of covariates and estimate the probability that a fish will attempt to escape. We additionally proposed to better examine the escapement process at the individual level with regard to the temporal dynamics of escapement over time. Our approach was used to analyze gadoids swimming and escapement behaviors collected using a video set up in front of a selective device known to improve selectivity on gadoids in the extension of a bottom trawl. Comparison of the fit of models indicates that i) the instantaneous rate of escape attempts is constant over time and that the escapement process can be modelled using an exponential law; ii) the mean time before attempting to escape increases with the increasing number of attempts; iii) more than 80% of the gadoids attempted to escape through the selective device; and iv) the estimated probability of success was around 15%. Effects of covariates on the probability of success were investigated using binomial regression but none of them were significant. The data set collected is insufficient to make general statements, and further observations are required to properly investigate the effect of intrinsic and extrinsic factors governing gadoids behavior in trawls. This methodology could be used to better characterize the underlying behavioral process of fish in other parts of a bottom trawl or in relation to other fishing gears.

## Introduction

The 14^th^ Sustainable Development Goal of UNESCO [1] emphasized the necessity to maintain a good ecological status in fish populations and their habitats. In Europe, the Common Fisheries Policy (CFP) focuses on minimizing the effects of fishing on ecosystem functioning while maintaining incomes of coastal communities. Since the beginning of 2019, this regulation prohibits the widespread practice of throwing back into the sea unwanted catches of stocks under total allowable catch (TAC) regulations (or with a Minimum Conservation Reference Size–MCRS–in the Mediterranean) [2]. As a result, most European fleets need to reduce their discards to maintain their fishing opportunities. Changes in fishing gear selectivity and spatiotemporal fishing strategies are the two main strategies that fishers can use to achieve this goal. In order to avoid undesired fish mortality on the deck of fishing vessels, unwanted catches should be avoided in the first place, either by preventing individuals entering the gear or by allowing escapement. Better designed fishing gears could help to improve the match between the fishers’ target species and those actually caught by the gears [3]. A large amount of literature has been dedicated to gear selectivity trials over the last two decades (for reviews see [4–6]), in which catches are compared between a standard gear used by fishers and a test gear to assess gains and losses in terms of size selectivity.

Knowledge on animal behavior is a key element to understand the capture processes of fishing gears [7, 8] and is useful for modifying fishing gear design with the objective of influencing catch species composition. As behavioral responses are species specific, with clear differences between flatfish and roundfish, but also between roundfish species, due to differences in swimming capacities and anti-predator strategies [9], numerous case specific studies were carried out. Research on the behavioral response of fish to longline gear [10], nets [11], and baited fish pots [12–14] has proved to be a fruitful way to assess and modify gear design. For towed gear such as trawls, specific emphasis on escapement to improve gear design and selectivity has been examined, especially for fish and squid in the mouth [15, 16] or cod, haddock, whiting and hake in the extension and codend [9, 17–21]. In addition to catch comparison experiment, underwater video could help identifying the differences between selective devices efficiency [22], by analyzing escapement attempts, successes and failures. However, the comprehension of underlying behavioral processes at individual and collective levels is still challenging.

The rapid expansion of the use of underwater cameras has facilitated unprecedented opportunities for studying the behavior of species interacting with fishing gears in their natural environment. Pioneer work qualitatively described fish behavior interacting with fishing gears [7]. Further research expand this fundamental knowledge by characterizing typical swimming behavioral responses in trawls such as cruising behavior in front of the mouth of the net [23], optomotor and herding behavior [20], horizontal and vertical distribution of individual in the net [15]. Fine scale descriptions of escapement behavior through the mesh were also provided, with for example active escaping fish approached a mesh at right angles by swimming straight ahead with very little change in direction, while other fish that approached the net at obtuse angles retreated by turning sharply [24].Then came count data and percentages. For example, the proportion of individuals entering the extension which escaped through the meshes were determined [19, 25] and used to compare extension and codend configurations [25]. More sophisticated statistical analysis of video footages are under development in marine surveys field. Using deep learning to identify and quantify fish as they pass through the net [26], proposed to calculate absolute abundance estimate in trawls. A method for computing volumetric fish density using stereo cameras was also recently published by [27]. However, technological advance in camera systems would greatly benefit from the parallel development of dedicated methodologies to studies fish behavior in interaction with fishing gears.

The quantification of fish behavior based on video observations suffers of several bias related to instrumental, environmental and behavioral events. First, the camera’s field of view does not cover the entire surface of the net or escapement device. Second, environmental conditions such as mud plume or strong turbidity can reduce image quality. Finally, fish can swim during different periods within the camera’s field. These features induce varying observation periods between individuals, which biases the estimation of behavior duration, comparison between fish and preclude for direct estimation of the probability of escapement. For example, it is not because an individual does not attempt to escape during its observation period that it will not attempt later on in time or in rear part of the gear. In this work, we develop a methodological framework which takes into account these specificities of the dataset to better understand the behavioral processes that underpins escapement processes in a trawl. We propose to describe the process of escapement attempts through time using a parametric Weibull mixture model. This survival model enables handling for right-censored observations, as well as testing effects of covariates and estimating the probability that a fish will attempt to escape.

We use behavioral descriptors combined with statistical tests and a parametric model to i) describe swimming behavior of gadoids in the extension, ii) estimate the probability of making an escapement attempt (volunteer contact of the snout with the net), and iii) estimate the probability of success of escapement attempts when these fish are in the extension. To go further, we propose to explore the temporal dynamics of escapements as well as potential influencing factors to infer the underlying behavioral processes. This methodological framework can help address specific questions such as: Does contact probability and the probability of escape success change over time between the beginning and the middle of the towing period? Do these probabilities evolve with the number of attempts? Are escapement attempts made preferentially in a specific location of the cylinder?

We illustrate our approach by analyzing data collected with a video camera located in front of a selective device, mainly consisting of a 100 mm square mesh in a specific location of the trawl extension. Numerous trials have been made to increase the number of escape opportunities in this part of the gear, including the fitting of different types of netting (larger mesh size, smaller hanging ratio or alternative mesh shapes). Depending on the species being targeted, the panels or sections can be placed in the upper, side, or lower parts of the extension and can also extend across the full circumference [28, 29]. There has been a particular focus on square mesh because it facilitates escapement by maintaining open mesh geometry compared with diamond mesh, which tends to close with towing tension [30]. The effectiveness of square mesh panels has been demonstrated in gadoid-directed fisheries as well as *Nephrops*-directed fisheries [31–35].

## Material and methods

### Experimental setup and data collection

**Fig 1:**
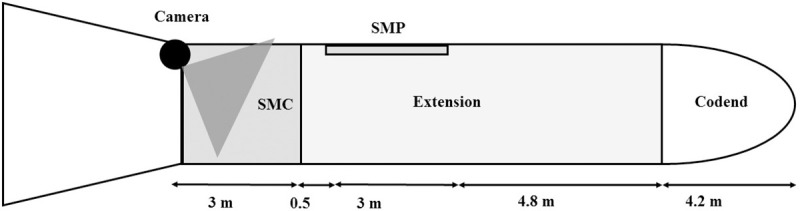
Schematic drawing of of the experimental extension and codend. The black circle represents the video camera and the grey triangle its field of view. SMC: Square mesh cylinder; SMP: Square mesh panel.

Experiments at sea were carried out onboard a commercial fishing vessel (22.85 m long, 497 kW and a gauge of 118.77 gt). The extension and codend of the trawl were diamond meshes made of 5 mm single TPE, with a nominal stretched-mesh size of 100 mm. The mandatory 100-mm square mesh panel (SMP) of 50 meshes long and 25 meshes wide was placed in the extension, 4.8 m before the codend. The additional square mesh cylinder (SMC) was made of two SMP seamed side by side to form a cylinder. At the junction between the SMC and the diamond mesh codend, each square mesh was seamed with two diamond meshes. The additional SMC was inserted prior to the SMP (Fig 1).

**Fig 2:**
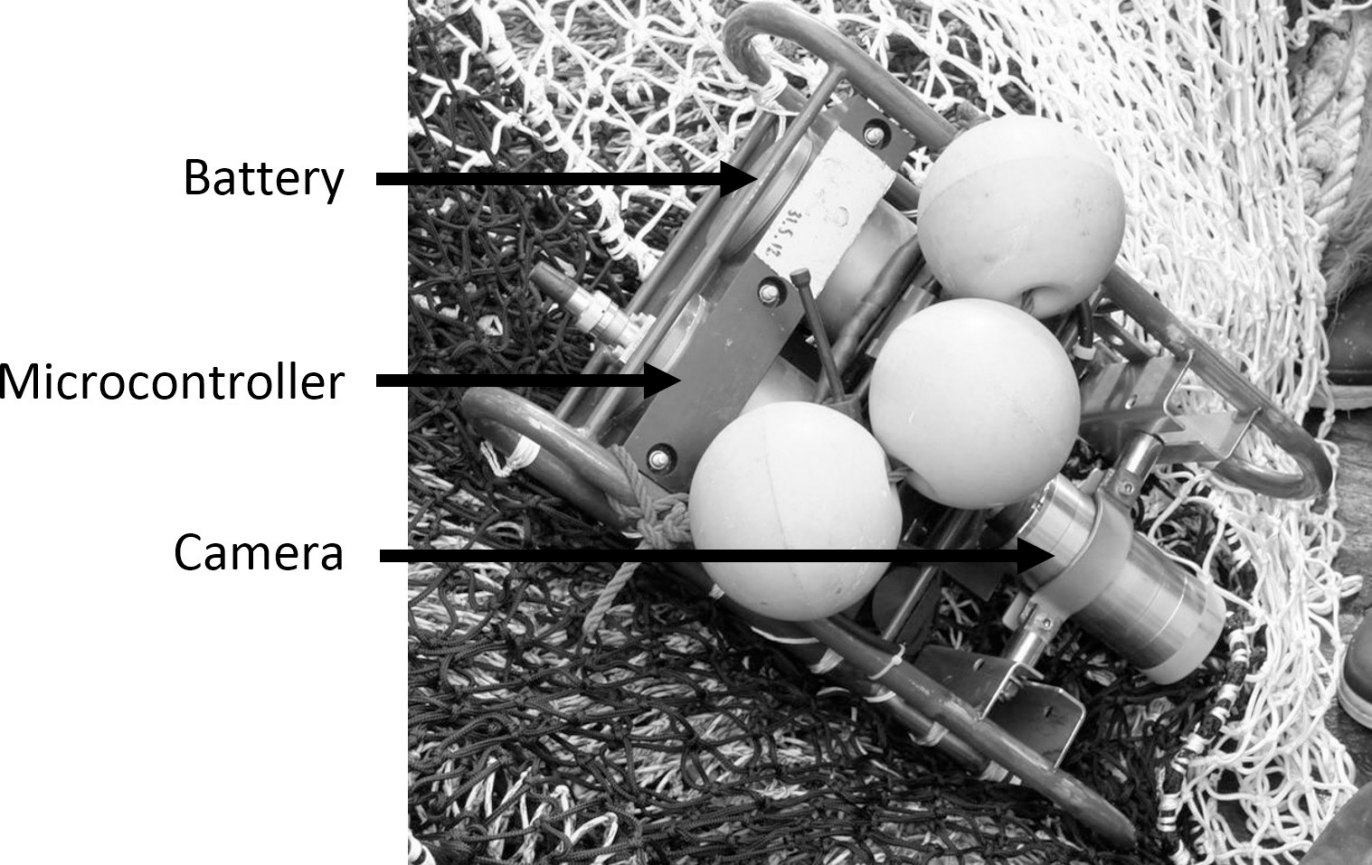
Photograph of the underwater video system, composed of three modules: Battery, microcontroller/memory and camera (Tornado low-light camera–Tritech, resolution = 570 TVL, S = 0.0003 lux, SNR > 50 dB).

One tow of approximately three hours was filmed underwater in the Celtic sea on September 13 2014, during the day at a depth of 123 m and a towing speed of 3 knots (initial coordinates of the tow 49°09’95 N– 07°00’74 W). The video camera system used was VECOC (Video Embarquée de Controle et d’Observation de Chalut), capable of generating black and white images under low-light conditions. The underwater video system was composed of three modules (Fig 2): battery, microcontroller/memory and camera (Tornado low-light camera–Tritech, resolution = 570 TVL, S = 0.0003 lux, SNR > 50 dB). The battery and microcontroller were housed in 10-cm diameter titanium pressure housings. Despite the camera sensitivity, a custom-made white LED light was used as a source of artificial light to assist the underwater camera.

The video system was placed inside the square mesh cylinder, in the upper part, facing toward the codend (Fig 1). The horizontal angle of the camera made it possible to analyze fish escaping the SMC from both the upper and lower parts. Two sequences of 5 min were analyzed, recorded at an interval of 50 min to test the influence of towing duration. The first sequence was started 10 min after the beginning of the tow. These sequences of video footage were selected for their good video quality and fish abundance.

### Methodological framework

#### Description of fish swimming behavior

The description of fish behavior was recorded using BORIS Software (Behavioral Observation Research Interactive [36]). Each fish entering the camera’s field of view was identified and individually followed until its disappearance from the field of view. Species were identified to the lowest taxonomic level possible. Swimming behavior was described using the position, orientation and speed of the fish. Four speed categories were estimated based on the relative position of the fish with respect to the trawl: i) the individual was not moving (‘immobility’), ii) the individual was swimming slower than the trawl (‘slow’), iii) swimming at the same speed as the trawl (‘medium’), or iv) swimming faster than the trawl (‘fast’). Fish position was categorized vertically (‘top’, ‘center’, ‘bottom’) and horizontally (‘left’, ‘center’, ‘right’). The left-right position was based on the movement direction of the trawl (i.e., the left is observed at the right of the video). The orientation of each fish’s body in the water column was defined in relation to the trawl axis: ‘forward’, ‘lateral’ (side-on) and ‘aft’. Individual swimming behaviors in the extension were characterized using a time budget: the percentage of time each fish spent in each position and orientation and at each speed.

Two metrics were recorded to describe escapement behavior: the escapement attempts and the success/failure of each attempt. An escapement attempt refers to voluntary swimming behavior toward the net where the snout then comes into contact with the net.

#### Escapement attempts

We used a parametric Weibull mixture distribution model [37, 38] to model the escapement process and estimate the probability of escapement attempts through time. This model formulation makes it possible to take into account several specificities of the studied process (i.e. the escapement attempt) and the type of observations. The observation period varied between individuals and the attempt could have occurred outside the field of the camera. As such, individuals that do not attempt to escape during the observation period can be considered and incorporated in the analysis as right-censored observations. Furthermore, the mixture distribution model allows that an unknown proportion of individuals do not attempt to escape. The resulting survival function S(t), i.e., the complementary cumulative distribution of escapement attempt events through time, is expressed as follows:

$$S\left(t\right)=P\left(T>t\right)=1‐\pi +\pi \;exp\left(‐{\left(\alpha t\right)}^{ɣ}\right)$$ (1)

Where T is the time elapsed between the arrival of a fish in the field of the camera and the event, or the time between two events. α > 0 and ɣ > 0 are the scale and shape parameters, respectively. The escapement attempt rate is expected to decrease with time and converge to an asymptote, 1 - π.

Compared to standard survival models, where the event is mortality, individual fish can attempt to escape several times. The time to escape and its rank was recorded for each attempt. Obviously, time was reset to zero after the first, second, and third attempts etc. The rank of the escapement attempts (first attempts, second attempts, etc.) of the same individual as well as the period of the tow when the video footage was recorded (beginning: video 1 or middle: video 2) were tested as categorical covariates on the three parameters (α, ɣ and π). Shape parameter was also fixed at 1 to test a simpler model formulation (the exponential model being a particular case of the Weibull model). This resulted in 32 potential models. Model parameters were estimated by maximization of the model likelihood using a quasi-Newton optimization algorithm. Models were ranked according to Akaike’s Information Criterion (AIC), which balances the goodness of fit by the number of parameters to favor model parsimony.

#### Probability of success/failure of escapement attempts

The effects of rank of escapement attempts and the period of video recording during the tow (video 1 or video 2) were tested on probability of success of escapement attempts using binomial GLM. Again, the model with the lowest AIC was selected.

#### Temporal dynamics of escapements

Results on the characterization of the escapement process at the individual level were examined with regard to the temporal dynamics of escapement over time by looking at the cumulative number of escapement attempts through time.

Statistical analyses were performed using R software. The minimal dataset is provided as a S1 File.

### Ethics statement

Observations were carried out on board the vessel Le Jusant in accordance with the European scientific fishing authorization granted by the French maritime fisheries and aquaculture directorate (2014/898456/SELECMCTrawl/0011 and 2014/898456/SELECMCTrawl/0002). The study is based on underwater video observation, a non-invasive method to study the behavior of fish in the water. Animals were not exposed to additional stress other than that involved in commercial fishing practice. Thus, no additional authorization or ethics approval was required to perform the study. This study did not involve endangered or protected species.

## Results

Haddock was by far the predominant species in the catches, with smaller numbers of whiting encountered (157 kg of haddock versus 17 kg of whiting). It was not always possible to distinguish between the two species. The two fish species were analyzed jointly and grouped under gadoids (as in [17]). Over the 10 min of video footage analyzed, 204 gadoids were recorded (106 and 98 for videos 1 and 2, respectively). The mean observation period on each fish was of 2.23 seconds (sd = 2.5, min = 0.28; max = 22.52).

### Swimming behavior

**Fig 3:**
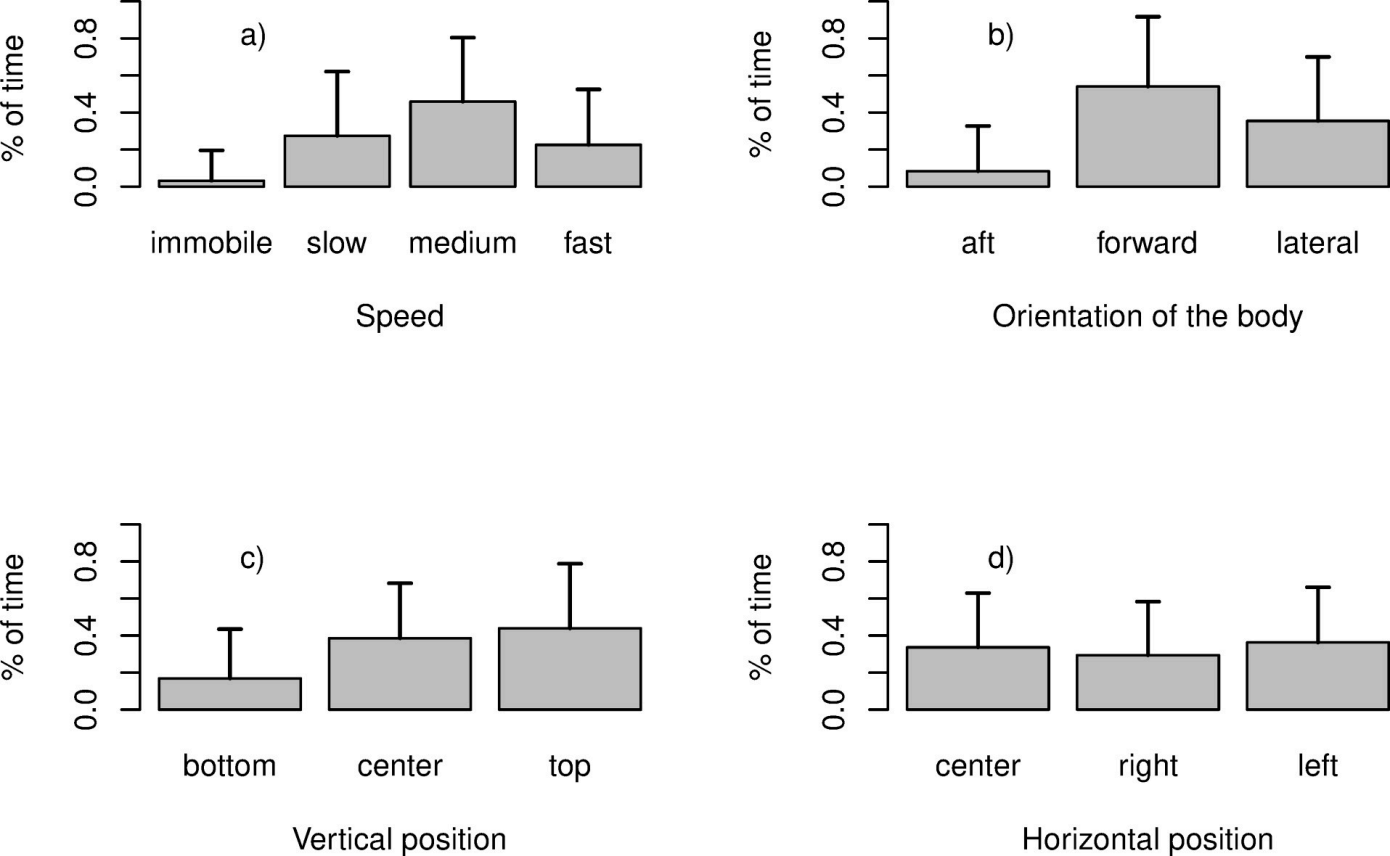
Bar plot of the percentage of time spent in each speed category a); with each body orientation b); in each horizontal and vertical position (c, d). Vertical bars represent standard deviations.

Time budget distribution in the 204 individuals observed is presented in Fig 3. The gadoids spent the majority of their time swimming at the same speed as the trawl but were observed to swim faster or slower than the trawl more than 20% of their time. The gadoids were predominantly oriented against the direction of current flow, in forward or lateral positions (Fig 3). Only 15% of the fish were oriented aft. They predominantly occupied the upper and central parts of the trawl cylinder while swimming, with these positions accounting for more than 80% of their time. They tended to occupy the horizontal space randomly (Fig 3D). These swimming characteristics were similar between the two periods of video footage recorded after one hour or two hours of towing (S1 Fig).

High variability observed in Fig 3 corresponds to strong inter-individual variability in swimming behavior. Indeed, the gadoids remained active in this part of the gear, with most showing frequent changes in direction or swimming speed.

### Escapement behavior

**Fig 4:**
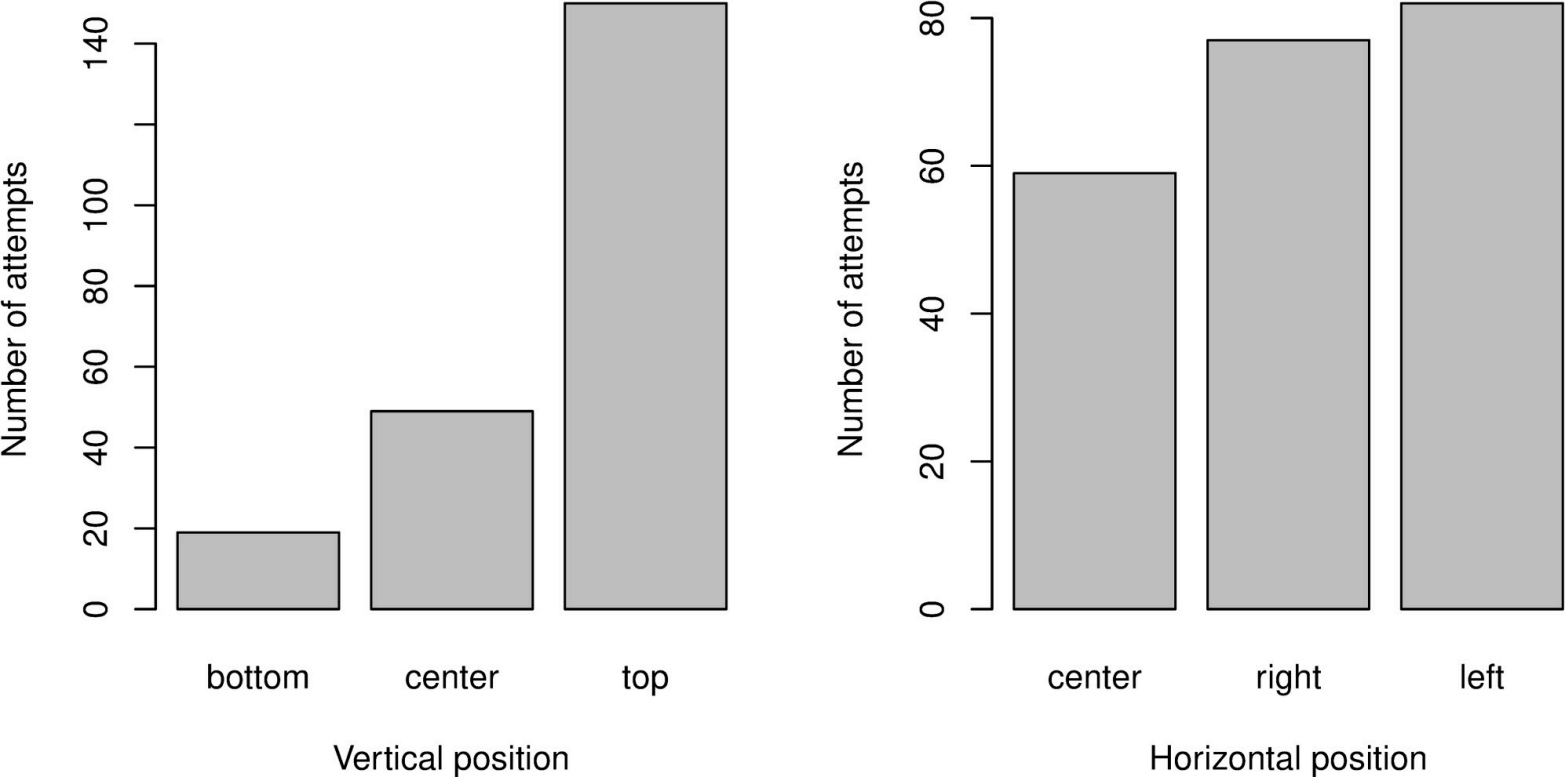
Horizontal and vertical positions of escapement attempts.

Out of the 204 individuals, 123 fish attempted to escape at least once, which represents 60% of the observed population. Two hundred and one escapement attempts were observed, meaning that the same individual could try to escape several times. On average, each fish that attempted to escape tried 1.77 times (sd = 1.15, min = 1, max = 6). Escapement attempts were not evenly distributed among positions in the gear: 69% of the attempts were made on the upper part of the net, while no differences were observed in horizontal positions (chi^2^, p-value = 0.13, Fig 4). These results remained similar between the two periods of video footage analyzed (S2 Fig). The time elapsed before making an escape attempt was 1.14 s on average (sd = 1.54, min = 0 and max = 13).

#### Escapement attempts

**Fig 5:**
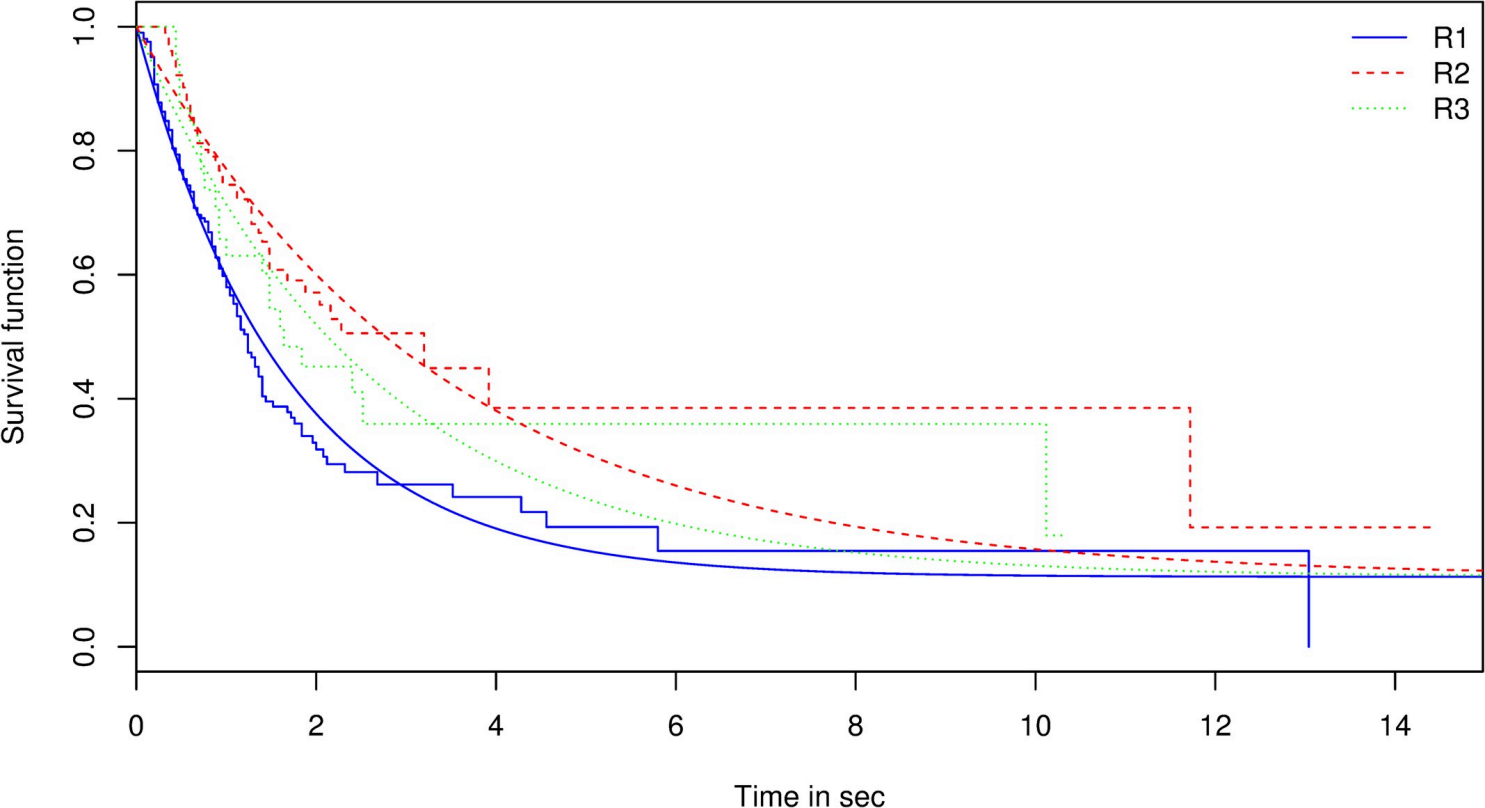
Kaplan Meir and adjusted survival functions for the first escapement attempt (solid blue line); second escapement attempt (dashed red line) and the first and subsequent escapement attempts (green dotted line).

The best-fitted model with the lowest AIC was model 8 in which ɣ was set at 1, corresponding to an exponential decay (Table 1). This means that the underlying behavioral process is stationary or, in other words, that the instantaneous rate of escapement attempts is constant over time. The best-fitting model also indicates that α differs between the first, second and third (and more) escapement attempts (α_R1 = 0.6, α_R2 = 0.29, α_R3+ = 0.38, Fig 4). The mean time before an escapement attempt (1/α) tended to increase between the first and second attempts. The proportion of the population that attempted to escape was estimated at 88% and did not depend on the rank of the attempt. Fig 5 illustrates how all curves converge to the same estimate of 1-π. The time of the video recording in the tow (video 1 vs video 2) was not retained by the model selection procedure for any of the three parameters tested.

**Table 1:** Escapement probability.

Model	Parameters on which the covariates are tested		AIC
	Rank of escape	Video	
M0	α, ɣ, π		750.23
M1	α, ɣ		747.79
M2	α, π		747.92
M3	ɣ, π		749.97
M4	α		744.83
M5	ɣ		757.58
M6	π		748.14
M7	α,π,ɣ = 1		745.92
M8	α,ɣ = 1		**742.83**
M9	π,ɣ = 1		746.14
M10			750.52
M11	ɣ = 1		753.43
M12		α, ɣ, π	760.28
M13		α, ɣ	758.42
M14		α, π	758.31
M15		ɣ, π	758.53
M16		α	756.48
M17		ɣ	757.41
M18		π	756.54
M19		α, ɣ = 1, π	756.31
M20		α, ɣ = 1	754.48
M21		ɣ = 1, π	754.54
M22	α, ɣ, π	α, ɣ, π	759.94
M23	α, ɣ	α, ɣ	754.89
M24	α, π	α, π	754.65
M25	ɣ, π	ɣ, π	756.53
M26	α	α	748.18
M27	ɣ	ɣ	760.84
M28	π	π	751.69
M29	α, ɣ = 1, π	α, ɣ = 1, π	752.65
M30	α, ɣ = 1	α, ɣ = 1	746.18
M31	ɣ = 1, π	ɣ = 1, π	749.69

The effect of the two covariates rank of the escape attempt and timing of the video footage (video 1 or 2) were tested on each parameter (α, ɣ, π) of the Weibull mixture model, resulting in 32 model configurations (M0 to M31). AIC estimates used for model selection are given with the AIC criterion for the selected model given in bold.

#### Success probability of escapement attempts

Comparison of goodness of fit indicated that the null model had the lowest AIC (Table 2). The probability of success of an escapement attempt did not depend on the rank of the attempt, or the period of the tow when the video recording was made (video 1: beginning or video 2: middle). The probability of success was estimated at 14.8%.

**Table 2:** Probability of success.

Binomial GLM	AIC
Rank+ Video+ Irreg_Chalut	40.04
Rank+ Video	38.2
Rank+Irreg_Chalut	38.05
Video+Irreg_Chalut	39.61
Rank	36.21
Video	37.82
Irreg_Chalut	37.61
**Null**	**35.83**

The effect of the two covariates: Rank of the escape attempt and timing of the video footage (video 1 or 2) were tested on the probability of escape using a binomial GLM, resulting in 4 model configurations. AIC estimates used for model selection are given with the AIC criterion for the selected model given in bold.

#### Temporal dynamics of escapement

**Fig 6:**
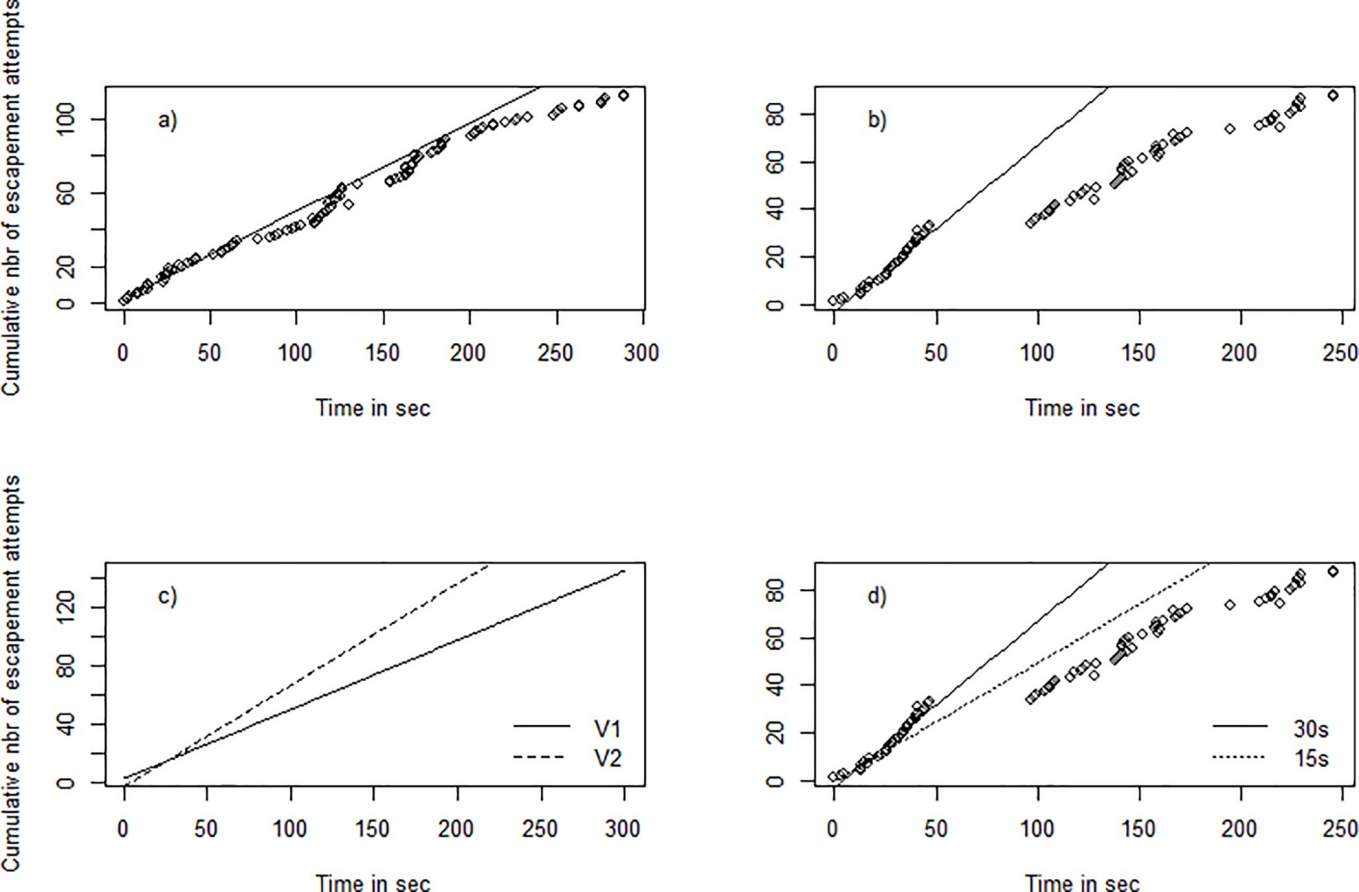
Cumulative number of escapement attempts through time (5 minutes) in video 1 (a) and video 2 (b) taken after an interval of 50 minutes (after 10 minutes and 1 h of towing, respectively). Solid lines represent linear regressions fitted on the first 30 s of observation of each video footage. Panel c) describes the linear regression adjusted on the first 30 s of videos 1 and 2. Panel d) illustrates the linear regression adjusted on the first 15 or 30 s of video 2.

If the underlying behavioral process driving escapement behavior at the individual scale can be modeled using an exponential process (constant hazard rate) and fish enter the trawl following a homogeneous Poisson process (purely random), the cumulative number of escapement attempts through time should follow a linear trend. Fig 6 illustrates the temporal dynamics of the escapement attempts over the 5 minutes of observation in videos 1 and 2 and a linear regression adjusted on the first 30 seconds of each video (see also the regression adjusted on the first 15 seconds of video 2, Fig 6D). One can see that the slopes of the linear regressions are quite similar between the two videos (Fig 6C). However, our results indicate that observations deviated from the linear regression, which suggests that social phenomena could be at work.

## Discussion

The use of an underwater video cameras allows direct observation of animal swimming behavior and for the two processes, the escapement attempt and its potential success, to be distinguished. Indeed, understanding the reasons for failure is just as important as the reasons for success. Despite various constraints such as video quality, water turbidity, and the time required to process video footage [39], numerous studies have used video to characterize animal behaviors or their interactions with fishing gears and especially with trawls (grid [25, 40–42]; drop chain [16, 43], trawl mouth [44, 45]; panel [46, 47]).

The data set analyzed (n = 204 gadoid fish individually followed over 10 minutes of observation from one fishing operation using artificial white light) allows to demonstrate the great potential of the methodological framework proposed to infer on swimming and escapement behavior of gadoids interacting with a 100 mm square mesh cylinder in the extension of a bottom trawl. Nevertheless, this data set was illustrative and not sufficient to make general statements from the results obtained.

Artificial lights is known to affect fish behavioral responses in trawls (reviewed by [48]). Although studies just ignore the bias, others use catch data in addition to the camera observations to determine if artificial lights affected fish behavior [49]. The use of artificial white light was required given the limited ambient light at 120-m depth in the Celtic sea. Due to limitations in our experimental design, we were unable to measure the effect of artificial light in our study. Several studies recommend using infrared light instead to minimize the impact of light on fish behavior [50, 51]. However, a limitation of using red and far red light is the high absorption of these wavelengths by the water. Nevertheless, it remains interesting to confront results of this pilot study to literature.

### Swimming behavior

Fish position in a trawl varies between species. Previous investigation of species-specific behavior in the aft and codend of a trawl have shown that Nephrops and flatfish remain low in the net, cod have a more uniform vertical distribution, and haddock and whiting stay high [21, 23]. This is in line with our observation of haddock and whiting swimming mainly in the central to upper part of the trawl in the towing direction.

The tendency of roundfish to show varying degrees of “rise” as they tire has led to the development of an upper panel in the extension to increase escapement rate of undersize fish [20, 52, 53]. Since 2017, a square mesh panel is mandatory in the Celtic sea to reduce fishing mortality of juvenile roundish (mainly haddock and whiting). Our observations confirm these previous findings and show that 70% of escapement occurred in the upper part of the trawl. Nevertheless, around 20% of escapement attempts took place through the side of the cylinder. As already pointed out by [34] for hake, increasing the surface of a selective device such as a square mesh device from the top to the sides or even to the bottom (to produce a cylinder) can increase the selectivity of several species.

Two types of swimming behavior are reported in the literature, 1) ordered and steady swimming behavior and 2) erratic swimming behavior characterized by a higher variation in angular velocity and faster swimming speed [54]. Our observations indicate that under the conditions of the experiment most of the gadoids were still actively swimming against the current flow, holding their position in relation to the camera (medium to fast swimming, positioned in a forward body orientation) when in the former part of the extension. This finding agrees with previous observations where optomotor responses were the most common behavior [19].

### Escapement behavior

#### Escapement attempts

At an average towing speed of 3 knots and given the swimming abilities of gadoids, escapement attempts in the extension must be an active and voluntary behavior [33]. In comparison [19], reported that in half of the observations in the codend, fish appear to come into contact with the net through the pulsing motion of the codend itself rather than actively swimming toward the netting, leading to “opportunistic escape”. The percentage (π) estimated in this pilot study (88%) appears higher than those reported for hake (62%) or megrim (41%) [34]. This difference could be attributed to the effect of artificial light used in our experiment (see above). However, it is worth noting that our approach provides an estimation of the expected percentage of the population that attempts to escape in the extension based on the temporal dynamic of the observed attempts, while in most of the published works it is reported the observed percentage in the view of the camera.

We also observed that haddock and whiting can make successive attempts to escape and that the mean time before an attempt increases between the first and second attempt and decreases between the second and third or more attempts. More observations should be made to confirm this pattern and better understand this behavioral phenomena (reinforcement, learning, etc.), as well as potential biases linked to the presence of light. Our modeling approach also allows testing whether π depends on the rank of escapement attempt. Our preliminary results suggest independence between successive attempts. The value of this parameter is likely to be species and size specific and might be influenced by net geometry.

Our results suggest little difference between the two video footages made at 50 min interval, suggesting that similar processes are at work in both. The hypothesis that the fish would become more tired as towing time increases and make less escapement attempts in the extension should be further investigated but is not supported by our observations.

By using curve fitting, we went further than studies in the literature have previously done. We gained knowledge on the underlying behavioral processes involved in escapement of fish in trawls. Our preliminary results seems to indicate that an escapement attempt is a memoryless phenomenon (ɣ = 1): the probability of attempts to escape can be modelled using an exponential law and the instantaneous rate of escapement is constant over time at the scale of 5 min. This type finding will be useful for improving hypotheses made to model fish behavior in trawls [55]. On the contrary, if additional observations indicated that in reality ɣ <1, this would indicate that the instantaneous rate of escapement decreases over time.

#### Escapement success

We estimate the probability of success of an escape attempt through a square mesh of 100 mm in the extension at 14.8% based on our experiment. This is lower than previously published results for the same species in the codend, where 20 and 35% of the fish approaching or striking the netting, respectively, resulted in successful escapes [19]. Escapement success is a size-related tradeoff: on the one hand, individuals that are too small, while capable of passing through the mesh easily, have poor swimming abilities and, on the other hand, individuals that are too large, while capable of good swimming performances, have too broad a cross section to penetrate through the mesh and escape. As such, the difference observed can result from difference in fish size (15-44cm in [19] compared to 25-65cm in our experiment, information coming from catch sampling). However, precise determination of fish length was not possible here using only one camera. Further studies with video observation of fish interacting with selective devices will require the development of a stereovision system to accurately quantify fish length, position and orientation [56].

It is reasonable to assume that fish encountered fishing gear on more than one occasion and that their behavior may be modified through a process of learning from past experiences [8] and that this could have implication for fisheries management [57]. Experiments run in laboratory conditions have demonstrated the role of learning in mesh penetration by haddock [58] and clupeoids [59]. However, how rapid can fish learn? The binomal regression we used allows testing such processes by testing the influence of successive attempts on the probability of success. Preliminary results suggest that it is unlikely that fish learn from recent failures, although additional research will be required, as it might vary with species and individuals.

In our experiment, the probability of success of observed attempts was the same after 10 minutes as after one hour of towing. In comparison, escapement from the codend evolves with time as catch build up there and obstruct the open mesh [19, 60].

Escape probability and its success can be more efficient for groups [18, 23]. Indeed, social behaviors for predator avoidance have been demonstrated in schooling fish [61], but social interactions and facilitations need to be further investigated in demersal fish communities. Applied to fishing gear [62], demonstrated that density of fish ahead of the bottom-trawl positively affects catchability and that qualitative differences can be observed in escapement and capture behavior at various densities [8]. More than density, social learning (e.g process by which individuals acquire new behavior via the observation or interaction with other animals [63]) among fish in response to approaching net was also demonstrated under laboratory conditions [63]. Results of our analysis on the dynamics of escapement attempts through time raise several questions and hypotheses. Our preliminary observations show that the cumulative number of escapement attempts over time does not strictly follow a linear trend. Two main explanations involving social behavior can be put forward to explain such differences. Firstly, fish might not be entering the extension following a homogeneous Poisson process. Indeed, when examining the total number of gadoids observed per second (S3 Fig) we can see that this is not statistically constant but evolved through time. This arrival of fish in pulses can induce small accelerations in the temporal dynamics. In this case, the temporal dynamic of escapements should be analyzed conditionally to arrivals and further methodological developments are required to combine both processes. Secondly, social interactions can play a role and influence temporal dynamics with, for instance, an effect of the number of congeners on following behavior in escapement, or the total number of fish through a crowding effect. When such interactions occur, the individual probability of escaping can increase with the number of fish. Further observations, coupled with modeling work, could help improve understanding of any underlying social mechanisms [64].

In conclusion, knowledge on fish behavior in fishing gear is still scarce and sometimes contradictory about the same device or species due to the influence of intrinsic (physiological condition, motivation state, fish size and visual ability) and extrinsic (ambient light levels, temperature, fish density) factors [8, 65]. This advocates for a deeper examination of capture and escapement underlying behavioral processes using laboratory studies, observations at sea and modelling. In this study, we develop a methodological framework including Weibull modelling which allows to take into account some specificities of the data set (variable observation periods between individuals, censored and recurrent measurement) and estimate parameters such as: the proportion of the population that tend to escape, the instantaneous rate of escapement and its temporal dynamics as well as effect of covariates on this parameters. We additionally proposed to better examine the escapement process at the individual level with regard to the temporal dynamics of escapement over time. This methodology is applied to the case study of gadoids escapement behavior faced with a selective device (100mm squared mesh cylinder) in the extension of a bottom trawl. Nevertheless, the results presented herein are based on a too small number of observations (in terms of fish number and number of fishing operation) to make general statements and additional research will be required to validate the findings.

## Supporting information

S1 Fig Time budget comparison between the two video recordings. (DOCX)

S2 Fig Comparison of escapement attempt location between the two video recordings. (DOCX)

S3 Fig Number of gadoids observed in the field of view per second in video 1(a) and video 2(b).
(DOCX)

S1 File (DOCX)
