# Supplementary figures and images for: A methodological framework for characterizing fish swimming and escapement behaviors in trawls
Source: PLoS One. 2020 Dec 11;15(12):e0243311. doi: 10.1371/journal.pone.0243311 (PMC7732098; doi:10.1371/journal.pone.0243311)

S1 Fig. Time budget comparison between the two video recordings


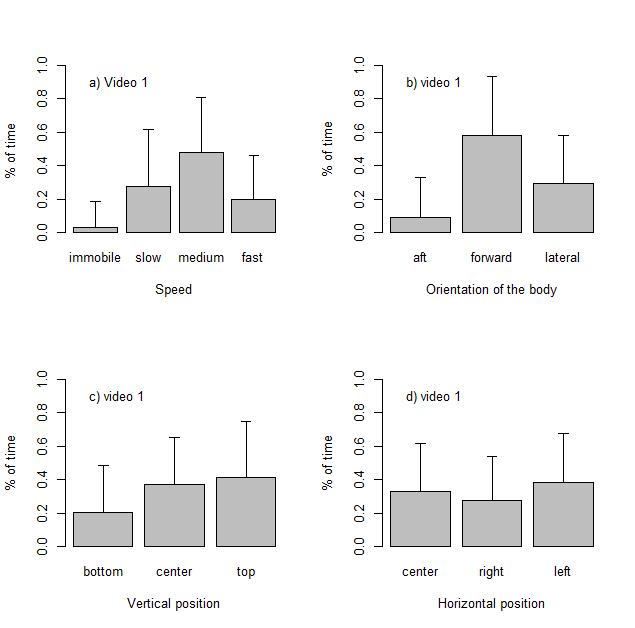


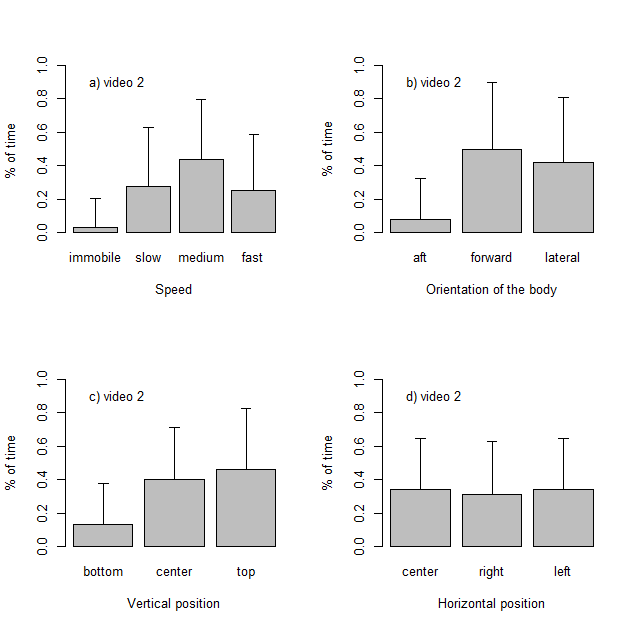

Supplement: S1 Fig — (DOCX) [file pone.0243311.s001.docx]

S2 Fig. Comparison of escapement attempt location between the two video recordings


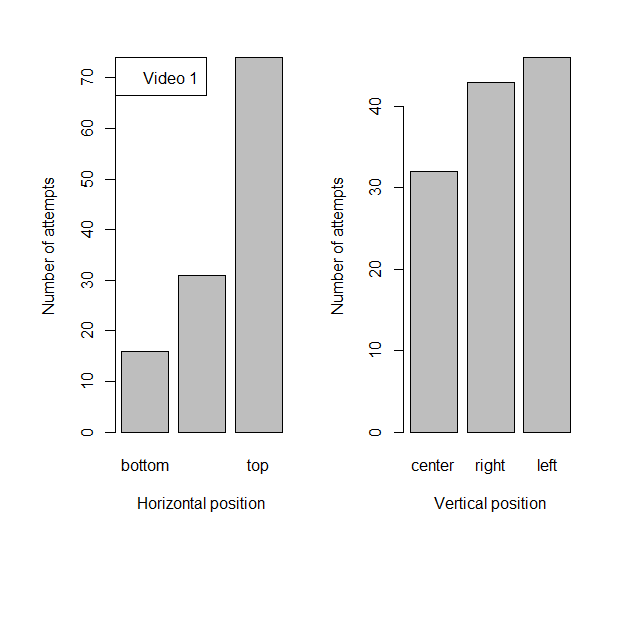

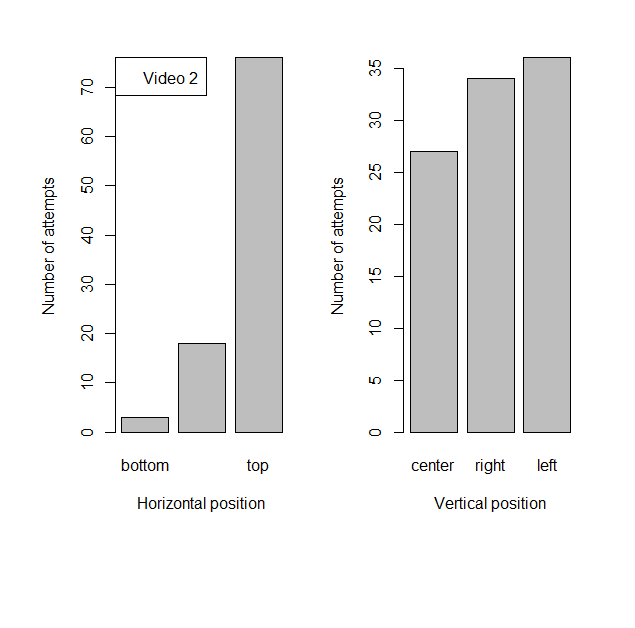

Supplement: S2 Fig — (DOCX) [file pone.0243311.s002.docx]

S3 Fig. Number of gadoids observed in the field of view per second in video 1(a) and video 2(b).


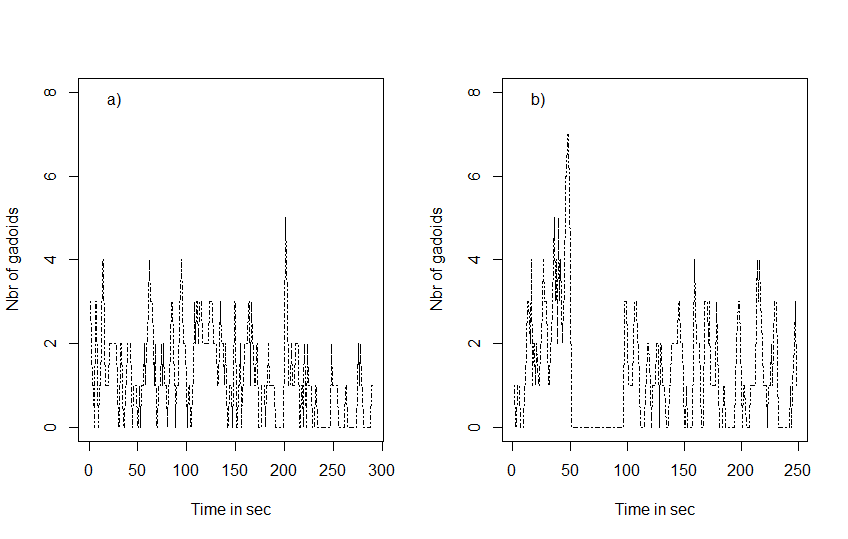

Supplement: S3 Fig — Number of gadoids observed in the field of view per second in video 1(a) and video 2(b). (DOCX) [file pone.0243311.s003.docx]
