# Supplementary material for: A methodological framework for characterizing fish swimming and escapement behaviors in trawls
Source: PLoS One. 2020 Dec 11;15(12):e0243311. doi: 10.1371/journal.pone.0243311 (PMC7732098; doi:10.1371/journal.pone.0243311)
Supplement: S1 File — (DOCX) [file pone.0243311.s004.docx]

Swimming behavior

|  | Percentage | SD |
| --- | --- | --- |
| Speed |  |  |
| immobile | 0.03 | 0.16 |
| slow | 0.27 | 0.35 |
| medium | 0.46 | 0.35 |
| fast | 0.23 | 0.30 |
| Body Orientation |  |  |
| aft | 0.08 | 0.24 |
| forward | 0.54 | 0.38 |
| lateral | 0.35 | 0.35 |
| vertical position |  |  |
| Bottom | 0.17 | 0.27 |
| center | 0.39 | 0.30 |
| top | 0.44 | 0.35 |
| horizontal position |  |  |
| center | 0.34 | 0.29 |
| right | 0.29 | 0.29 |
| left | 0.36 | 0.30 |

Escapement attempts: Weibull model

| Time to escape (in sec) | Censured | Video | Rank of escapement |
| --- | --- | --- | --- |
| 2.2 | 0 | 1 | R1 |
| 1.32 | 0 | 1 | R1 |
| 0.92 | 0 | 1 | R1 |
| 1 | 0 | 1 | R1 |
| 0.52 | 0 | 1 | R1 |
| 1.24 | 0 | 1 | R1 |
| 0.92 | 0 | 1 | R1 |
| 0.52 | 0 | 1 | R1 |
| 2.32 | 0 | 1 | R1 |
| 2.04 | 0 | 1 | R1 |
| 5.68 | 0 | 1 | R1 |
| 1.92 | 0 | 1 | R1 |
| 3.56 | 0 | 1 | R1 |
| 0.88 | 0 | 1 | R1 |
| 1.52 | 0 | 1 | R1 |
| 0.56 | 0 | 1 | R1 |
| 1 | 0 | 1 | R1 |
| 2.36 | 0 | 1 | R1 |
| 1 | 0 | 1 | R1 |
| 3.68 | 0 | 1 | R1 |
| 1.64 | 0 | 1 | R1 |
| 1 | 0 | 1 | R1 |
| 1 | 0 | 1 | R1 |
| 1.24 | 0 | 1 | R1 |
| 0.56 | 0 | 1 | R1 |
| 0.84 | 0 | 1 | R1 |
| 2.52 | 0 | 1 | R1 |
| 1.2 | 0 | 1 | R1 |
| 1.04 | 0 | 1 | R1 |
| 2.36 | 0 | 1 | R1 |
| 1.96 | 0 | 1 | R1 |
| 0.72 | 0 | 1 | R1 |
| 1.92 | 0 | 1 | R1 |
| 1.04 | 0 | 1 | R1 |
| 0.4 | 0 | 1 | R1 |
| 0.6 | 0 | 1 | R1 |
| 1.72 | 0 | 1 | R1 |
| 1.16 | 0 | 2 | R1 |
| 4.72 | 0 | 2 | R1 |
| 0.56 | 0 | 2 | R1 |
| 0.76 | 0 | 2 | R1 |
| 0.76 | 0 | 2 | R1 |
| 4.96 | 0 | 2 | R1 |
| 0.64 | 0 | 2 | R1 |
| 1.12 | 0 | 2 | R1 |
| 6.2 | 0 | 2 | R1 |
| 0.72 | 0 | 2 | R1 |
| 6.84 | 0 | 2 | R1 |
| 7.12 | 0 | 2 | R1 |
| 2.64 | 0 | 2 | R1 |
| 2.64 | 0 | 2 | R1 |
| 0.64 | 0 | 2 | R1 |
| 1 | 0 | 2 | R1 |
| 1.12 | 0 | 2 | R1 |
| 0.76 | 0 | 2 | R1 |
| 1.76 | 0 | 2 | R1 |
| 0.64 | 0 | 2 | R1 |
| 1.04 | 0 | 2 | R1 |
| 1.12 | 0 | 2 | R1 |
| 1.36 | 0 | 2 | R1 |
| 0.64 | 0 | 2 | R1 |
| 1.36 | 0 | 2 | R1 |
| 0.28 | 0 | 2 | R1 |
| 1.48 | 0 | 2 | R1 |
| 2.32 | 0 | 2 | R1 |
| 2 | 0 | 2 | R1 |
| 1.72 | 0 | 2 | R1 |
| 0.76 | 0 | 2 | R1 |
| 1.12 | 0 | 2 | R1 |
| 2.52 | 0 | 2 | R1 |
| 1.64 | 0 | 2 | R1 |
| 0.84 | 0 | 2 | R1 |
| 0.64 | 0 | 2 | R1 |
| 0.6 | 0 | 2 | R1 |
| 1.76 | 0 | 2 | R1 |
| 1.12 | 0 | 2 | R1 |
| 1 | 0 | 2 | R1 |
| 0.76 | 0 | 2 | R1 |
| 2.12 | 0 | 2 | R1 |
| 1.6 | 0 | 2 | R1 |
| 0.56 | 0 | 2 | R1 |
| 0.28 | 1 | 1 | R1 |
| 1.16 | 1 | 1 | R1 |
| 1.32 | 1 | 1 | R1 |
| 0.84 | 1 | 1 | R1 |
| 0.08 | 1 | 1 | R1 |
| 1.04 | 1 | 1 | R1 |
| 0.52 | 1 | 1 | R1 |
| 5.8 | 1 | 1 | R1 |
| 1.4 | 1 | 1 | R1 |
| 0.32 | 1 | 1 | R1 |
| 1.84 | 1 | 1 | R1 |
| 2.68 | 1 | 1 | R1 |
| 0.16 | 1 | 1 | R1 |
| 0 | 1 | 1 | R1 |
| 1.52 | 1 | 1 | R1 |
| 0.64 | 1 | 1 | R1 |
| 0.44 | 1 | 1 | R1 |
| 3.52 | 1 | 1 | R1 |
| 0.2 | 1 | 1 | R1 |
| 0.68 | 1 | 1 | R1 |
| 0.4 | 1 | 1 | R1 |
| 0.8 | 1 | 1 | R1 |
| 1.72 | 1 | 1 | R1 |
| 1.08 | 1 | 1 | R1 |
| 4.28 | 1 | 1 | R1 |
| 0.24 | 1 | 1 | R1 |
| 1.16 | 1 | 1 | R1 |
| 1.12 | 1 | 1 | R1 |
| 0.36 | 1 | 1 | R1 |
| 1.84 | 1 | 1 | R1 |
| 0.84 | 1 | 1 | R1 |
| 1.36 | 1 | 1 | R1 |
| 1.4 | 1 | 1 | R1 |
| 0.56 | 1 | 1 | R1 |
| 0.48 | 1 | 1 | R1 |
| 1.32 | 1 | 1 | R1 |
| 0 | 1 | 1 | R1 |
| 1 | 1 | 1 | R1 |
| 0.32 | 1 | 1 | R1 |
| 0.64 | 1 | 1 | R1 |
| 0.88 | 1 | 1 | R1 |
| 1.08 | 1 | 1 | R1 |
| 0.48 | 1 | 1 | R1 |
| 1.16 | 1 | 1 | R1 |
| 0.4 | 1 | 1 | R1 |
| 0.72 | 1 | 1 | R1 |
| 0.64 | 1 | 1 | R1 |
| 0.36 | 1 | 1 | R1 |
| 0.2 | 1 | 1 | R1 |
| 0.16 | 1 | 1 | R1 |
| 0.12 | 1 | 1 | R1 |
| 0.68 | 1 | 1 | R1 |
| 1.24 | 1 | 1 | R1 |
| 0.96 | 1 | 1 | R1 |
| 1.2 | 1 | 1 | R1 |
| 1.4 | 1 | 1 | R1 |
| 0.6 | 1 | 1 | R1 |
| 4.56 | 1 | 1 | R1 |
| 0.76 | 1 | 1 | R1 |
| 0.64 | 1 | 1 | R1 |
| 1.96 | 1 | 1 | R1 |
| 0.16 | 1 | 1 | R1 |
| 0.96 | 1 | 1 | R1 |
| 0.24 | 1 | 1 | R1 |
| 0.48 | 1 | 1 | R1 |
| 1.12 | 1 | 1 | R1 |
| 1.24 | 1 | 1 | R1 |
| 1 | 1 | 1 | R1 |
| 0.4 | 1 | 1 | R1 |
| 0.4 | 1 | 2 | R1 |
| 0.52 | 1 | 2 | R1 |
| 0.84 | 1 | 2 | R1 |
| 0.88 | 1 | 2 | R1 |
| 1.36 | 1 | 2 | R1 |
| 0.24 | 1 | 2 | R1 |
| 0.48 | 1 | 2 | R1 |
| 0.36 | 1 | 2 | R1 |
| 0.2 | 1 | 2 | R1 |
| 0.88 | 1 | 2 | R1 |
| 0.84 | 1 | 2 | R1 |
| 0.8 | 1 | 2 | R1 |
| 0.56 | 1 | 2 | R1 |
| 0.24 | 1 | 2 | R1 |
| 0.4 | 1 | 2 | R1 |
| 1.44 | 1 | 2 | R1 |
| 0.48 | 1 | 2 | R1 |
| 0.2 | 1 | 2 | R1 |
| 0.16 | 1 | 2 | R1 |
| 1.12 | 1 | 2 | R1 |
| 0.64 | 1 | 2 | R1 |
| 1 | 1 | 2 | R1 |
| 0.16 | 1 | 2 | R1 |
| 1.4 | 1 | 2 | R1 |
| 0.2 | 1 | 2 | R1 |
| 0.08 | 1 | 2 | R1 |
| 0.92 | 1 | 2 | R1 |
| 0.32 | 1 | 2 | R1 |
| 2.12 | 1 | 2 | R1 |
| 0.44 | 1 | 2 | R1 |
| 1.24 | 1 | 2 | R1 |
| 0.92 | 1 | 2 | R1 |
| 0.2 | 1 | 2 | R1 |
| 2.32 | 1 | 2 | R1 |
| 0.28 | 1 | 2 | R1 |
| 0.2 | 1 | 2 | R1 |
| 1.68 | 1 | 2 | R1 |
| 0.6 | 1 | 2 | R1 |
| 0.2 | 1 | 2 | R1 |
| 2.08 | 1 | 2 | R1 |
| 0.92 | 1 | 2 | R1 |
| 13.04 | 1 | 2 | R1 |
| 1.24 | 1 | 2 | R1 |
| 1.28 | 1 | 2 | R1 |
| 2 | 1 | 2 | R1 |
| 0.2 | 1 | 2 | R1 |
| 0.28 | 1 | 2 | R1 |
| 1.76 | 1 | 2 | R1 |
| 0.52 | 1 | 2 | R1 |
| 0.24 | 1 | 2 | R1 |
| 0.8 | 1 | 2 | R1 |
| 0.4 | 1 | 2 | R1 |
| 1.04 | 1 | 2 | R1 |
| 0.24 | 1 | 2 | R1 |
| 1.32 | 0 | 1 | R2 |
| 1.2 | 0 | 1 | R2 |
| 2.84 | 0 | 1 | R2 |
| 2.12 | 0 | 1 | R2 |
| 3.16 | 0 | 1 | R2 |
| 0.76 | 0 | 1 | R2 |
| 1.24 | 0 | 1 | R2 |
| 2.84 | 0 | 1 | R2 |
| 8.28 | 0 | 1 | R2 |
| 2.4 | 0 | 1 | R2 |
| 2.48 | 0 | 1 | R2 |
| 3.08 | 0 | 1 | R2 |
| 1.76 | 0 | 1 | R2 |
| 5.28 | 0 | 1 | R2 |
| 1.4 | 0 | 1 | R2 |
| 0.72 | 0 | 1 | R2 |
| 2.12 | 0 | 1 | R2 |
| 1.8 | 0 | 1 | R2 |
| 1.12 | 0 | 1 | R2 |
| 1.44 | 0 | 1 | R2 |
| 1.24 | 0 | 1 | R2 |
| 1.24 | 0 | 1 | R2 |
| 1.68 | 0 | 1 | R2 |
| 1.48 | 0 | 1 | R2 |
| 2.32 | 0 | 1 | R2 |
| 0.76 | 0 | 1 | R2 |
| 1.2 | 0 | 1 | R2 |
| 3.44 | 0 | 1 | R2 |
| 2.52 | 0 | 1 | R2 |
| 0.72 | 0 | 1 | R2 |
| 0.76 | 0 | 1 | R2 |
| 1.6 | 0 | 1 | R2 |
| 0.84 | 0 | 2 | R2 |
| 1.64 | 0 | 2 | R2 |
| 1.84 | 0 | 2 | R2 |
| 1.08 | 0 | 2 | R2 |
| 2.28 | 0 | 2 | R2 |
| 1.44 | 0 | 2 | R2 |
| 0.64 | 0 | 2 | R2 |
| 1.2 | 0 | 2 | R2 |
| 2.84 | 0 | 2 | R2 |
| 0.6 | 0 | 2 | R2 |
| 2.64 | 0 | 2 | R2 |
| 2.12 | 0 | 2 | R2 |
| 0.64 | 0 | 2 | R2 |
| 1.72 | 0 | 2 | R2 |
| 1.12 | 0 | 2 | R2 |
| 5.04 | 0 | 2 | R2 |
| 14.4 | 0 | 2 | R2 |
| 2.12 | 0 | 2 | R2 |
| 2.48 | 0 | 2 | R2 |
| 6.04 | 0 | 2 | R2 |
| 0.56 | 0 | 2 | R2 |
| 0.8 | 0 | 2 | R2 |
| 2.6 | 0 | 2 | R2 |
| 0.88 | 0 | 2 | R2 |
| 1.28 | 0 | 2 | R2 |
| 1.6 | 0 | 2 | R2 |
| 1.24 | 0 | 2 | R2 |
| 0.6 | 1 | 1 | R2 |
| 0.44 | 1 | 1 | R2 |
| 1.28 | 1 | 1 | R2 |
| 0.92 | 1 | 1 | R2 |
| 0.64 | 1 | 1 | R2 |
| 1.479 | 1 | 1 | R2 |
| 1.68 | 1 | 1 | R2 |
| 2.04 | 1 | 1 | R2 |
| 0.88 | 1 | 1 | R2 |
| 0.64 | 1 | 1 | R2 |
| 0.68 | 1 | 1 | R2 |
| 1.24 | 1 | 1 | R2 |
| 0.44 | 1 | 1 | R2 |
| 0.56 | 1 | 1 | R2 |
| 0.56 | 1 | 1 | R2 |
| 3.2 | 1 | 1 | R2 |
| 0.6 | 1 | 1 | R2 |
| 1.48 | 1 | 1 | R2 |
| 0.8 | 1 | 1 | R2 |
| 0.76 | 1 | 1 | R2 |
| 2.16 | 1 | 1 | R2 |
| 0.36 | 1 | 1 | R2 |
| 0.44 | 1 | 1 | R2 |
| 0.4 | 1 | 1 | R2 |
| 1.88 | 1 | 2 | R2 |
| 0.36 | 1 | 2 | R2 |
| 3.92 | 1 | 2 | R2 |
| 0.68 | 1 | 2 | R2 |
| 1.48 | 1 | 2 | R2 |
| 0.56 | 1 | 2 | R2 |
| 1.28 | 1 | 2 | R2 |
| 1.12 | 1 | 2 | R2 |
| 1.4 | 1 | 2 | R2 |
| 1.12 | 1 | 2 | R2 |
| 11.72 | 1 | 2 | R2 |
| 0.52 | 1 | 2 | R2 |
| 0.96 | 1 | 2 | R2 |
| 0.36 | 1 | 2 | R2 |
| 1.36 | 1 | 2 | R2 |
| 0.32 | 1 | 2 | R2 |
| 0.96 | 1 | 2 | R2 |
| 2.28 | 1 | 2 | R2 |
| 0.52 | 1 | 2 | R2 |
| 7.56 | 0 | 1 | R3 |
| 2.4 | 0 | 1 | R3 |
| 10.36 | 0 | 1 | R3 |
| 1.56 | 0 | 1 | R3 |
| 1.72 | 0 | 1 | R3 |
| 2.2 | 0 | 1 | R3 |
| 2.72 | 0 | 1 | R3 |
| 2 | 0 | 1 | R3 |
| 0.96 | 0 | 1 | R3 |
| 2.92 | 0 | 2 | R3 |
| 2.16 | 0 | 2 | R3 |
| 2.51 | 0 | 2 | R3 |
| 1.08 | 0 | 2 | R3 |
| 3.84 | 0 | 2 | R3 |
| 2.8 | 0 | 2 | R3 |
| 0.92 | 1 | 1 | R3 |
| 0.44 | 1 | 1 | R3 |
| 0.72 | 1 | 1 | R3 |
| 0.76 | 1 | 1 | R3 |
| 1.64 | 1 | 1 | R3 |
| 10.12 | 1 | 1 | R3 |
| 2.4 | 1 | 1 | R3 |
| 1 | 1 | 1 | R3 |
| 0.48 | 1 | 1 | R3 |
| 1.48 | 1 | 1 | R3 |
| 0.72 | 1 | 1 | R3 |
| 0.44 | 1 | 1 | R3 |
| 0.88 | 1 | 1 | R3 |
| 0.68 | 1 | 1 | R3 |
| 1.6 | 1 | 2 | R3 |
| 0.6 | 1 | 2 | R3 |
| 0.48 | 1 | 2 | R3 |
| 2.52 | 1 | 2 | R3 |
| 1.84 | 1 | 2 | R3 |
| 0.48 | 1 | 2 | R3 |
| 1.4 | 1 | 2 | R3 |
| 0.92 | 1 | 2 | R3 |
| 1.48 | 1 | 2 | R3 |

Probability of success of observed attempts: Binomial model

| Rank of escapement | Video | Nbr of failure | Nbr of success |
| --- | --- | --- | --- |
| 1 | 1 | 44 | 8 |
| 1 | 1 | 12 | 5 |
| 1 | 2 | 35 | 8 |
| 1 | 2 | 11 | 0 |
| 2 | 1 | 14 | 1 |
| 2 | 1 | 9 | 0 |
| 2 | 2 | 12 | 3 |
| 2 | 2 | 3 | 1 |
| 3 | 1 | 10 | 2 |
| 3 | 1 | 2 | 0 |
| 3 | 2 | 5 | 0 |
| 3 | 2 | 4 | 0 |
| 4 | 1 | 2 | 0 |
| 4 | 1 | 2 | 0 |
| 4 | 2 | 3 | 0 |
| 4 | 2 | 1 | 0 |
| 5 | 1 | 2 | 0 |
| 5 | 2 | 1 | 0 |
| 5 | 2 | 1 | 0 |
